# The Bacilli of Syphilis and Hydrargyrum Tannicum Oxydulatum*Read before the Chicago Medical Society, January 19, 1884.

**Published:** 1885-02

**Authors:** Joseph Zeisler

**Affiliations:** 125 State Street; Chicago


### Article II.

### The Bacilli of Syphilis and Hydrargyrum Tannicum Oxydulatum.* *By* Dr. Joseph Zeisler, *Chicago.*

*Mr. President and Gentlemen of the Society:*—At the meeting of this society on December 15th, I had the intention to inform you of a new and, as I think, important discovery, but the discussion of Dr. Murphy's paper took so much time that I had not the opportunity to do so. Since that meeting five weeks have passed, and I really do not know that I can give you anything new to-night, supposing that most of you have

*Read before the Chicago Medical Society, January 19, 1884.

read about it in medical journals. But I would not like to disappoint the programme of the evening, and so I shall make some remarks on the Bacillus of Syphilis.

At the meeting of the Vienna Medical Society on November 21st, Dr. Sigmund Lustgarten made a preliminary communication on specific Bacilli of Syphilis, and I think it best to give you a translation of it.

"The great importance of the subject, the peculiar character of the results obtained by me, as also the unanimous and concurrent opinion of eminent physicians, such as Koch and Weigert, and the desire to establish my claim to priority, have induced me, notwithstanding the small number of investigated cases, to give an account of the same.

"I have succeeded in showing, in microscopical sections of two syphilitic chancres and one syphiloma, bacilli, which are perfectly characterized by their color, reaction, form and relative position. These bacilli, which have been found in all of the examined sections, although in slightly varying quantity, represent slim, straight, or somewhat curved little rods of about the same size and the same appearance as the bacilli of tuberculosis. They are always found either single or in small groups, inclosed in lymphoid, somewhat distended cells, and show under a powerful microscope light spots similar to those which Koch regards as "spores" in the tubercle-bacilli. The method of coloring, about which I shall report in a future and more exhaustive paper, makes it possible to distinguish the bacilli of syphilis both from the bacilli of lepra and tuberculosis and from all other pathogenic bacteria as yet known. The fact that the fo[illegible] always are inclosed in cells excludes the possibility of deceptions by putrid formations, and so on. I never could observe anything like cocci, and I emphasize this, because a number of investigators (deceived by more or less important errors) have regarded them as specific micro-organisms of syphilis (as Birch-Hirschfeld, and others).

"I refrain, for the present, from speaking of any ætiological relation of the described bacilli to syphilis, only desiring to state the fact of my microscopical results."

Thus far Lustgarten.

Now, gentlemen, the mistakes of Finkler and Prior, in Bonn, in regard to the claimed identity of the bacilli of cholera nostras to cholera asiatica ought to be a warning example, to prevent rash conjectures. Likewise you certainly will remember that Lostorffer, in Vienna, about fifteen years ago, thought that he had found characteristic micro-organisms in the blood of syphilitic individuals, which he called "Syphilis Körperchen," corpuscles of syphilis, but which afterwards were found in lupus and even in normal persons.

But from long personal acquaintance, I know Dr. L. as a thoroughly scientific and very sceptical investigator; he has spent the greater part of the last year in the laboratory of Prof. Weigert, in Leipsic, and surely he has also made the pure cultures of the new bacillus; at all events the confirmation by the great master, Koch, is important enough to banish our doubts. As to the importance of the new discovery, I think you will agree with me that it perhaps exceeds that of the cholera bacillus, as syphilis is a disease of such general occurrence, and not of an epidemic character.

Dr. Sigmund Lustgarten has also introduced into the practice a new remedy against syphilis, *hydrargyrum tannicum oxydulatum.* It is nearly a year ago that the first trials with it were made at Prof. Kaposi's clinic for skin diseases, in Vienna, and the results showed, as you will see further on, that it was accompanied by some remarkable advantages. It is already quite often used in Germany, and Dr. Görges, in a paper on the present state of treatment of syphilis, read before the "Gesellschaft für Heilkunde," in Berlin, as well as Professor Auspitz, in his introductory lecture in his clinic for syphilis, in

Vienna, mentioned it as a very useful preparation. But as far as I could learn, till now, very little notice of it has been taken in this country, and Messrs. Shering and Glatz, in New York, who have the privilege of selling it in the United States, told me that as yet there has been no demand for it. I think, therefore, that a short reference to it and its properties will not be without interest to the profession.

It would surpass the limits of my paper to begin here with even a condensed review of the present state of the treatment of syphilis. Nor do I deem it necessary to state that mercury is now generally regarded as possessing specific effects against that disease. As to the different ways of introducing mercury into the human body, I think that the old method of frictions will still for a long time maintain its position and extended use, though now mostly regarded as quite unscientific and rather barbarous.

The great progress which has been made by the subcutaneous application of mercurial solutions is surely invaluable in spite of the disadvantages combined with it.

Still, there are very many cases where both methods are not practicable, and where we prefer the internal treatment. The largest use in this direction was made till now by the Protoioduretum of Hydrargyrum, first recommended by Ricord, by calomel and by sublimate. But all of them answer very unsatisfactorily the purpose which we expect and demand of them, that is, a rapid absorption into the circulation without disagreeable effect. I think, in both regards, the *hydrargyrum tannicum* is more valuable than all other preparations.

The remedy referred to is the tannate of the protoxide of mercury; it is a dull green powder without odor or taste, and is only soluble when decomposed. Hydrochloric acid makes no sensible impression upon it; by nitric acid it is easily dissolved with a brown color. Very dilute alkalies, ammonia, solution of caustic potash and the alkaline carbonates, reduce it in a short time to a sort of magma composed of very small particles of metallic mercury. They are so minute that the greater part of them present, under the microscope, the phenomenon known as molecular movement. This process of reduction is produced under the influence of the alkaline reaction of the intestinal juices, and it is more than probable that the absorption of the particles of mercury in the intestines is produced in a similar way as on the skin by mercurial frictions; it might also be regarded as an analogous to the absorption of fat, which also takes place after the fat has been transformed into a state of emulsion.

By all means the rapid resorption into the system is proved by an abundant mercurial precipitate in the urine short time after introducing it. This can easily be understood, as the new preparation contains about 50 per cent. of metallic mercury.

The remedy was tried in nearly all forms of syphilis, either recent eruptions or inveterate, in papular, pustular and gummous syphilides. Dr. Lustgarten has reported about twelve of these cases in Nos. 11 to 14 of the *Wiener Medizinische Wochenschrift;* I have seen all of them, being at that time at Kaposi's clinic as assistant. I can confirm that in all of them a rather surprising result was obtained, even in those cases of relapse of a very rebellious nature, as syphilides with small papules and pustules. Six of those cases were primary eruptions, among which two syphilides with small papules and one with large papules and pustules; three other cases were relapses of secondary syphilis, and three were gummous forms. In two of these latter cases local applications of mercurial plaster were made, according to the fact that in such cases local and constitutional treatment work together in harmony. But in all of the remaining cases no other remedy was prescribed, and sometimes when a patient had some experience in that disease, we prescribed frictions with an indifferent ointment, in order to detain him in the clinic for the purpose of study. Already seven to fourteen days after the preparation had been used, a rapid regression of the lesions ordinarily could be observed, and after three or four weeks all were cured.

In this city I had only one opportunity to use it. It was in a young man, H. G., about 25 years old. Chancre in last June; six weeks later roseola. He said that he had been treated by internal and external remedies, at last by zinc ointment (!). When I first saw him on Sept. 13th, I found a very largely diffused maculo-papular syphilide. I recommended mercurial frictions, and immediately wrote to New York to get the *hydrargyrum tannicum.* From September 19th until October 5th he took three decigrammes of the preparation every day; on the latter date the eruption had entirely disappeared; only a few pigment spots could be seen. No symptoms of mercurial intoxication could be observed. I ordered him to continue the use of the salt, and on November 2d I discharged him as cured.

The new drug has been prescribed in doses of one decigramme (about one and a half grains) with four parts of sugar, two to three times a day, one hour after meals; this latter in order to avoid direct irritation of the mucous membrane of the mouth. An addition of alkaline carbonates, as well as larger quantities of mineral waters containing such salts, should be avoided, as these would decompose the salt. Also the use of iodide of potassium is not good, because larger masses of iodide of hydrargyrum could be formed.

It is remarkable that disagreeable symptoms of mercurial intoxication—stomatitis and salivation—as so often seen in case of other preparations, never followed the use of this salt; only in one case could we see a swelling of the gums. A disagreeable influence on the intestines, the gravest of all objections to other internally used mercurial preparations, did not make itself felt, even when the hydrarg. tann. was taken for weeks, and in that seemingly large dose of four decigrammes daily. The digestion was always undisturbed; the action of the bowels regular. This can be explained partly from the decomposition of the salt by the intestinal juices, partly from the presence of tannic acid. Still, the hydrarg. tann. should only be given when all organs of digestion are in a normal condition. In order to avoid a possible irritation of the intestines, Lustgarten recommended to add to each dose five centigrammes of tannic acid, or one-half centigramme of opium.

I do not think the new drug will produce quite a revolution in the treatment of syphilis, but the effects as yet noticed are so remarkably good that the new method deserves to be placed by the side of the best methods hitherto employed. To treat that disease, I should be glad if some of you would try the drug and report about it at some future date. As I mentioned, it can be procured of Messrs. Shering & Glatz, in New York, and Mr. Sargent of this city has ordered it at my instigation.

125 State Street.

